# A Review of the Clinical Characteristics and Novel Molecular Subtypes of Endometrioid Ovarian Cancer

**DOI:** 10.3389/fonc.2021.668151

**Published:** 2021-06-03

**Authors:** Shuangfeng Chen, Yuebo Li, Lili Qian, Sisi Deng, Luwen Liu, Weihua Xiao, Ying Zhou

**Affiliations:** ^1^ Department of Obstetrics and Gynecology, Anhui Provincial Hospital, Anhui Medical University, Hefei, China; ^2^ Department of Obstetrics and Gynecology, The First Affiliated Hospital of USTC, Division of Life Sciences and Medicine, University of Science and Technology of China, Hefei, China; ^3^ Division of Molecular Medicine, Hefei National Laboratory for Physical Sciences at Microscale, The CAS Key Laboratory of Innate Immunity and Chronic Disease, School of Life Sciences, University of Science and Technology of China, Hefei, China; ^4^ Institute of Immunology, University of Science and Technology of China, Hefei, China

**Keywords:** microbiota dysbiosis, molecular subtypes, treatment strategy, prognosis, molecular characteristic, endometrioid ovarian cancer

## Abstract

Ovarian cancer is one of the most common gynecologic cancers that has the highest mortality rate. Endometrioid ovarian cancer, a distinct subtype of epithelial ovarian cancer, is associated with endometriosis and Lynch syndrome, and is often accompanied by synchronous endometrial carcinoma. In recent years, dysbiosis of the microbiota within the female reproductive tract has been suggested to be involved in the pathogenesis of endometrial cancer and ovarian cancer, with some specific pathogens exhibiting oncogenic having been found to contribute to cancer development. It has been shown that dysregulation of the microenvironment and accumulation of mutations are stimulatory factors in the progression of endometrioid ovarian carcinoma. This would be a potential therapeutic target in the future. Simultaneously, multiple studies have demonstrated the role of four molecular subtypes of endometrioid ovarian cancer, which are of particular importance in the prediction of prognosis. This literature review aims to compile the potential mechanisms of endometrioid ovarian cancer, molecular characteristics, and molecular pathological types that could potentially play a role in the prediction of prognosis, and the novel therapeutic strategies, providing some guidance for the stratified management of ovarian cancer.

## Introduction

Ovarian cancer (OC) is a global public health issue and threat to women’s health. According to the Global Cancer Statistics, which includes data of 36 cancers from 185 countries, nearly 300,000 new cases of OC were diagnosed worldwide in 2018 and 184,799 women died of OC in the same year (1). These figures make OC the third most diagnosed malignancy and the leading cause of death in female gynecological oncology. Epithelial ovarian cancer (EOC)is the most common and lethal type of OC, further divided into the high-grade serous, low-grade serous, endometrioid, clear cell, mucinous. Up to now, the study of EOC mainly focuses on high-grade serous carcinoma. With the development of precision medicine, the therapeutic direction of carcinoma has gradually turned to targeted and stratified therapy.

Endometrioid ovarian cancer (EOVC) accounts for 10~15.8% of EOC (2), and shows an association with endometriosis (3) and Lynch syndrome (4, 5). EOVC is often accompanied by synchronous endometrial carcinoma (EC) and typically diagnosed at an early stage (6). It is estimated that 84~95% of EOVC cases are of grades I and II, with grade III representing 5~16% (**Table 1**). More than 70% of EOVC are diagnosed at Federation International of Gynecology and Obstetrics (FIGO) I-II according to OC statistics in the United States in 2018 (7), and importantly, of high-grade EOVC that 65% were early stage (FIGO I/II) (8). However, some patients with EOVC still have a poor prognosis, and the proposed molecular classification may aid in providing an accurate prediction of the prognosis of patients with early-stage or low-grade EOVC, so as to better guide the clinical individualized treatment. The current pathogenesis of EOVC is also under discussion. This review introduces the origin, molecular characteristic, molecular classification, treatment and potential therapeutic strategies of EOVC and further explores factors influencing treatment choice and prognosis.

**Table 1:** Proportions of EOVC cases with different grades and stages.

Grade/FIGO stage	Proportion(%)
Grade	
Grade1/2	84~95%
Grade3	5~16%
Stage	
Stage I	50~72%
Stage II	11~36%
Stage III	12~14%
Stage IV	1~3%

## Epidemiology and Risk Factors

EOVC and ovarian clear cell carcinoma (OCCC) make up the second and third most common types of EOC, representing 20~40%. In Asia, there is a higher proportion of EOVC and OCCC and a lower proportion of serous carcinomas than other regions (9), however, the difference in distinct countries is still unclear. Multiple studies have shown that the following may increase the risk of developing EOVC: endometriosis; certain gene mutations; familial cancer syndrome (Lynch syndrome); disruption of the microbiota in the female reproductive system; age at menopause; body mass index (BMI) (10–15). Most risk factors show obvious heterogeneity in the five histologic subtypes of EOC, indicating different etiologies.

## Hypothesis of EOVC Origin

### Endometriosis

The most widely accepted hypothesis concerning endometriosis is the theory of retrograde menstruation, proposed by Sampson, who demonstrated that the shed endometrium can retrogradely enter the peritoneal cavity along the fallopian tube, and implant into the peritoneum and pelvic organs, including the ovaries, which can lay the foundations for the development of OC (16, 17).

Relevant articles have reported that approximately 25~80% of patients with EOVC and OCCC also have a diagnosis of endometriosis (18, 19). Patients with endometriosis have been reported to be at a 1.49, 3.73 and 2.32 times greater risk of development of OC, OCCC and EOVC respectively, compared with healthy women without endometriosis (20). In addition, approximately one third of endometrioid borderline ovarian tumors (EBOTs), are also associated with endometriosis (21). In recent years, whether or not endometriosis is a precancerous lesion of OC, in particular EOVC, has been a hot topic of concern for researchers.

A large number of studies have also found that patients with endometriosis have many gene mutations, including mutations in ARID1A, PIK3CA, KRAS, FBXW7, MLH1, ERBB2, CTNNB1, and PPP2R1A (19, 22, 23). Following comparison of mutations present in pure endometriosis and endometriosis-related OC, several population-based studies have suggested that endometriosis is a risk factor for OC. This may be due to the gradual development of ectopic endometrial tissue that engrafts onto the ovaries, along with sufficient driver mutations (20, 24). Of particular note, the ARID1A gene is thought to be involved in the progression of endometriosis to carcinoma (25, 26). The receptor activator of nuclear factor κ-B (RANK) signaling pathway has already been revealed to be involved in some tumor progression (27, 28), such as breast, bone, and lung cancers. Compared with the expression in the normal endometrium, expression of RANK is increased in patients with endometriotic lesions and EOVC (29).

Recently, an article defined “high-risk” cases as those which share the same mutations present in EOVC and endometriosis, and “low-risk” cases as those not sharing any mutations in endometriosis with the carcinoma (30). According to the literature, compared with women without endometriosis, women with endometriosis are more likely to be diagnosed with early-stage tumor, with a significantly lower level of serum CA125 before surgery, and are less likely to have lymph node metastasis or to develop platinum resistance disease (18, 31). Patients with EOVC arising from endometriosis presented at a lower average age, have a higher percentage of early-stage and lower–grade disease, and are more likely to have no residual disease after primary debulking surgery compared with those without endometriosis, which indicates better survival outcomes (32). EOVC arising from endometriosis was not statistically significant as an independent prognostic factor, therefore, endometriosis may be a possible precursor of EOVC but is not a factor that exacerbates cancer after its onset (33). Patients with endometriosis can be divided into several groups according to malignancy risk and there are schemes and principles used to categorize patients: ROMA (risk of ovarian malignancy algorithm), RMI (risk of malignancy index), and IOTA (International Ovarian Tumor Analysis simple rules) which are based on serum biomarkers, BMI and ultrasound findings (34).

### Microbial Communities in Female Reproductive Tract

**Figure 1:**
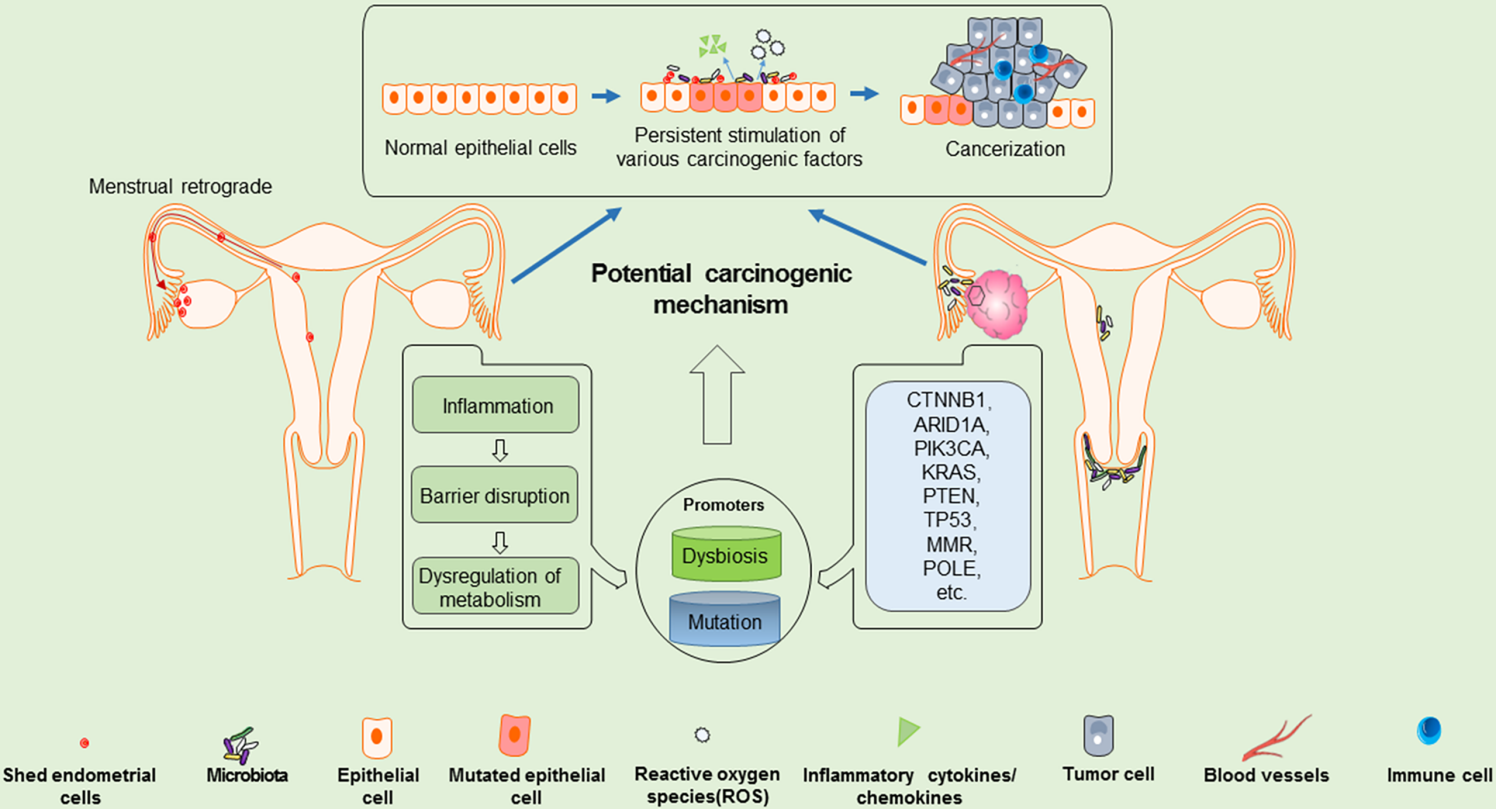
Potential carcinogenic mechanisms of EOVC: shed endometrial cells may migrate retrogradely into the ovary, which may be a pro-factor for EOVC. Along with, microenvironment dysbiosis and accumulation of mutation burden, the shed endometrial cells and ovarian epithelial cells may gradually evolve into atypical cells, and further transform into carcinoma.

It is generally believed that the upper reproductive tracts (i.e. the uterus, fallopian tubes, and ovaries) are sterile, however, recent reports on the microbiota of the female reproductive tract haves demonstrated that bacteria are present in the uterus and ovaries (35, 36), however, microbial abundance is relatively low compared with the vagina and cervix. Dysregulation of the vaginal environment is a risk factor for many diseases, as upward colonization of the reproductive tract with microbiota, especially anaerobic bacteria, serves as a primary driver for inflammation, and may be involved in the development of diseases, such as gynecological cancers (37–39). Where there is persistent dysbiosis of the microbial environment, altered immune and metabolic signaling can result in oxidative stress and the recruitment of immune cells which release reactive oxygen species (ROS), which may result in inflammation–driven carcinogenesis (40, 41). At present, the study of endometrial microbiota has reported that EC is associated with particular microorganisms (42). Similarly, related studies have also indicated that many microorganisms are involved in the development of EOC, such as Proteobacteria and Firmicutes (43). Brucella, Chlamydia, and Mycoplasma have been detected in over 60% of samples from those with EOC, and it is thought that the microbial dysbiosis may contribute to development of atypical epithelial cells showing hyperplasia and cytological atypia. These changes, along with genetic mutations in the ovaries gradually progress to EOC cells, particularly endometrioid and clear cell types (44–46). **Figure 1** illustrates the potential carcinogenic mechanisms involved in the development of EOVC. Sophisticated proteomic tracing studies suggest that EOVC arises from the secretory cells of the endometrium or endometriosis, while OCCC tends to arise from ciliated cells (47). It is hypothesized that the unique microenvironment dictates the development of ciliated or secretory cells, which then gain sufficient mutations to become malignant (48).

## Synchronous Ovarian and Endometrial Carcinoma

Some women are diagnosed with EC and EOVC at the same time and it can be arduous to identify whether they demonstrate metastasizing focuses of a single tumor or if they have synchronous but relative independent primary tumor (49). If it is a secondary tumor, there is a question over how metastasis occurred, from the endometrium to the ovary or from the ovary to the endometrium. Researchers have begun to explore this question, but opinions currently differ and there are still controversies.

There is evidence that low-grade EOVC and EC are similar in both molecular and histological characteristics (49, 50). By comparing the mutant spectra of EOVC and EC (**Table 2**), many shared mutant genes have been identified: ARID1A, TP53, PTEN, PIK3CA, KRAS, CTNNB1, MMR, POLE, among others (51). However, the frequency of mutation of these genes appears to vary depending on different microenvironmental effects (50–60). PTEN mutations are more frequent in low-grade endometrioid endometrial carcinoma (EEC) and CTNNB1 mutations are more common in low-grade EOVC (50). More patients with synchronous ovarian and uterine endometrioid carcinomas showed MLH1/PMS2 deficiency and PTEN aberrations compared with isolated EOVC (53).

**Table 2:** Summary of most common molecular alterations in EOVC and EEC(50-60).

Mutated genes	EOVC	EEC
ARID1A	30~55%	39~55%
TP53	6~26%	6~18%
PTEN	14~29%	64~80%
PIK3CA	15~43%	22~59%
KRAS	12~26%	19~43%
CTNNB1	16~63%	16~28%
MMR	7~18%	7~28%
POLE	3~10%	7~15%
FBXW7	13%	–
SOX8	19%	–
PPP2R1A	12~17%	–
ERBB2	8%	–

Synchronous tumors also have some commonly shared clinical characteristics: diagnosis tends to be at an earlier age and earlier stage, and prognosis is better compared with single primary ovarian or endometrial cancer (6, 61–63), although some studies have concluded that there is no difference in prognosis between a single primary carcinoma and synchronous tumors (64). One article has indicated that concurrent tumors are typically low-grade and confined to the uterine corpus and ovary, and that, despite being clonally related, behave much less aggressively than would be expected from a single advanced-stage cancer at either site (65). Moreover, a retrospective study comparing endometrial and ovarian synchronous primary cancers with ovarian metastases from EC, found that the synchronous primary cancer group typically had a history of endometriosis, the ovaries typically displayed a unilateral solid mass and the endometrial lesions were mostly non-vascularized; whereas the metastatic cancer group typically had bilateral solid ovarian masses (66). Synchronous EOVC and EC resulting from Lynch syndrome are concordant at a molecular level, suggesting a shared origins (67).

## Molecular Characteristics of EOVC

In terms of genomics, several unique heterogeneous genome profiles have been found in EOVC: CTNNB1, ARID1A, PIK3CA, KRAS, PTEN, TP53, MMR, POLE, SOX8, FBXW7, PPP2R1A, and ERBB2 (5, 68). A study investigating CTNNB1, a gene encoding β-Catenin protein, which participates in the Wnt signaling pathway, in EOVC patients showed that the rate of mutation is higher compared with that in EEC patients, and is associated with excellent clinical outcomes (69). This result contradicts other study findings in EEC which associate CTNNB1 mutations with a greater chance of recurrence (70). Mutation of the ARID1A gene, which is mainly involved in the formation of the SWI/SNF chromatin complex, may be an early event in the transformation of endometriosis into cancer (26, 71). Presence of ARID1A mutations and loss of the ARID1A-encoded protein BAF250a are frequently observed events in EOVC, presenting in 36~48% of cases (69, 72). PIK3CA mutation and/or amplification, linked with a low FIGO stage and low-grade, and are frequently observed in EOVC. The amplification of PIK3CA can weaken therapeutic response to chemotherapy, and may serve as a marker to predict response to chemotherapy in EOC (73, 74). KRAS mutations, which function to activate the MAPK pathway, play an important role in the development of endometriosis-associated cancer, including EOVC (75). PTEN loss is also a putative driver in EOC, and is associated with immunoresistance and poor response to programmed cell death protein 1 (PD-1) inhibitors. Downregulation of cytoplasmic PTEN expression is common in EOVC (76). Genomic data suggest that concurrent loss of PTEN and ARID1A with activating mutations of PIK3CA are involved in the pathogenesis of EOVC and OCCC. Abnormal expression of TP53 was also frequently seen in poorly differentiated endometrioid and clear cell tumors (77), being involved in several molecular subtypes of EOVC.

In addition to the more common genetic mutation events described above, mutations in mismatch repair (MMR) genes such as MLH1, MSH2, MSH6 and PMS2 form an important basis for the diagnosis of Lynch syndrome, Around 50% of cases of Lynch syndrome are diagnosed at the onset of EC and OC (78, 79), mainly endometrioid and clear cell carcinoma (80–82). Women with Lynch syndrome have a lifetime risk of developing ovarian and endometrial cancers of 5.8~12% and 40~62%, respectively (78, 83); with a further increased risk over the age of 40 (84). It has been reported in the literature that about 7~18% of cases of EOVC have MMR deficiency (69, 85), and this population also share similar characteristics: younger age (<50 years), higher CA125 at diagnosis, absence of ARID1A and higher FIGO stage (85, 86). Mutations of BRCA1/2 are well-known as the most frequent mutations to occur in OC, mainly in high-grade serous ovarian carcinoma (HGSOC); however, reports of BRCA1/2 mutations in individual EOVC cases are not common. A study of EOC in Australian showed that 10 of 119 (8.4%) women with EOVC have BRCA1/2 mutation and 8 of 10 EOVC were subsequently reclassified as serous or unspecified adenocarcinoma after strict histopathology review (87)

Although EOVC is less common than serous carcinoma, as an independent histological subtype, it is still difficult to diagnose clinically and is often confused with other types, such as HGSOC and mixed epithelial carcinoma. In particular, grade 3 EOVC may mimic HGSOC (88, 89). Therefore, knowing the gene mutation characteristics and whether cells have specific markers that other subtypes do not have will further help us to diagnose and study the disease (**Table 3**).

**Table 3:** The difference of immunohistochemistry (IHC) between EOVC and HGSOC in clinical cases.

Types	Proportion (%)	Tumor marker (determined by IHC)
EOVC	Most cases	WT1 (-); TP53 (wild-type); PR (+)/ER (+); Napsin-A (-)
	10~30%	WT1 (+) and/or P53 (abn ^a^ )
HGSOC	Most cases	WT1 (+); P53 (abn ^a^ ); p16 (+) ^b^

^a^null or >70% expression of TP53; ^b^diffuse expression.

In proteomics, distinct markers may distinguish EOVC from other histological types. Serous tumor markers such as WT1, P53, CK20, and mucinous tumor markers such as CEA and MUC2 are not often expressed in EOVC and OCCC, and EOVC–specific markers such as ER, PR, TFF3, DKK1, and MMP7 display near-exclusive expression in EOVC (90). Current studies have found that the status of WT1, P53, Napsin-A are helpful in reducing the rate of misdiagnosis of EOVC (88, 89, 91, 92). The vast majority of low-grade EOVC are WT1(-), P53(wild-type), ER(+) or PR(+), Napsin-A(-) (3, 89, 91, 93–95), and about 10~30% of EOVCs, especially high-grade EOVC, are WT1(+) or display abnormal P53 expression, which is easily confused with HGSOC (94, 96–98). Low-grade EOVC were characterized by strong nuclear β-catenin staining (99). Although controversial, high-grade EOVC may be considered to be a subtype of HGSOC according to immunophenotypic and gene profiling studies. In general, WT1 (+) and abnormal or absent P53 expression are highly suspicious for HGSOC (96, 100), but this does not mean that the classification is absolutely correct. Immunohistochemistry (IHC) is the only robust independent reference for OC histological subtypes (97).

## Molecular Typing of EOVC

**Figure 2:**
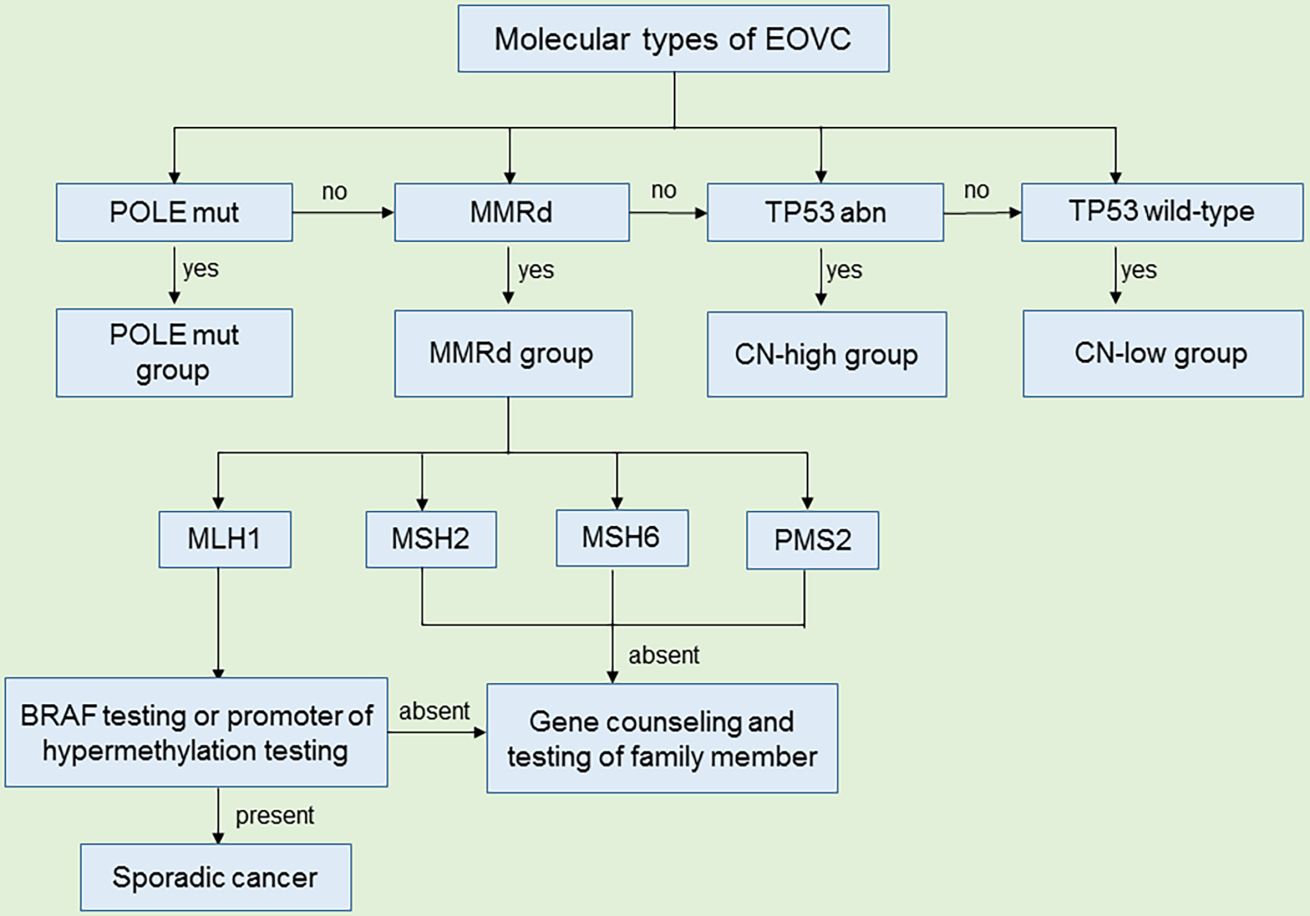
Criteria for molecular typing of EOVC (103). All EOVC patients were grouped according to POLEmut, MMRd, TP53abn and TP53wild-type. For patients with MMR protein deficiency, after excluding sporadic cancers, genetic counseling and testing of family members are required to prevent hereditary cancers. (mut, mutation; d, deficiency; abn, abnormal).

The molecular typing of EC has become a conventional tool in guiding treatment for individuals and in stratifying cases in clinical trials (101, 102). EOVC and EEC, have many similarities in their molecular characteristics and histology and share the same molecular types. According to IHC and next–generation sequencing (NGS) technology, EOVC is divided into the following four groups (96): TP53 wild-type (TP53wt) group with no obvious abnormalities, which accounts for the largest proportion (51.2~73.2%) of EOVC, followed by TP53 abnormal (TP53abn) group (9.6~24%), MMR protein deficiency (MMRd) group (8.3~19.4%), and the POLE hyper mutant (POLEmut) group (2.8~10%) (95, 96) (**Figure 2**). In addition, tumors of the POLEmut and MMRd groups were less frequent in EOVC compared with those in EC (95, 96, 101). The distinction in the proportion of molecular classifications also reflects different microenvironments to some extent.

### POLE Mutation

The POLE mutation is defined by pathogenic POLE exonuclease domain mutations that identify a group with an ultramutation phenotype. Somatic POLE exonuclease domain mutations occur early, quite possibly initiating events in sporadic cancers, and forcefully shape subsequent tumor evolution (104). Extreme genomic instability is characteristic of tumors with POLE mutations, with the mutation burden being among the highest found in human cancers. Tumors with POLE mutations display a distinct mutational signature, lymphocytic infiltrate and have an excellent prognosis. Although patients with POLE mutations in EOVC have high-risk pathological features, the prognosis is the same as that of EC, which further validates the feasibility of this classification in EOVC (105).

### MMR Protein Deficiency (MMRd) Group

The microsatellite instability (MSI) group is defined by deficiency of MMR (MLH1, MSH2, MSH6, or PMS2) proteins and identifies cases with microsatellite instability and a corresponding hypermutation phenotype. The MMRd group in EOVC is associated with a younger age and an increased number of tumor-infiltrating lymphocytes (4). The relationship between the MSI phenotype caused by the loss of MMR protein and Lynch syndrome has been clarified. This part of the population should be routinely screened for associated tumors to reduce the occurrence of non–ovarian malignant tumors (106).

### CN-High Group (TP53 Abnormal)

The CN-high group (TP53 abnormal) is correlated with a high copy-number genomic phenotype and abnormal TP53 IHC staining pattern. EOVC patients with TP53 abnormalities had a higher frequency of poorly differentiated cells (G2/G3) (95) compared with other molecular types and have the worst prognosis. Research has reported that EOVC with abnormal TP53 may be the result of CTNNB1 mutation (95).

### CN-Low Group (TP53 Wild-Type)

CN-low group (TP53 wild-type) comprises cases without any of the above three characteristics and is correlated with a copy-number low class. Research regarding the TP53wt group is relatively sparce due to its excellent survival state. In the context of TP53wt cases, in combination with CTNNB1 state (69), reveals that TP53wt (CTNNB1m) cases are almost always diagnosed at an early stage, rarely show macroscopic residual disease and have a low genomic complexity and low copy number alterations burden. However, the genomic complexity of TP53wt (CTNNB1wt) patients is relatively high compared with the TP53wt (CTNNB1m) group. Although the outcome of the TP53wt group is better, the difference in prognosis has not been fully explained and further study is still required.

**Table 4** summarizes the published literature on the application of molecular typing of EC and EOVC. All published articles are based on data from the centers in Europe and the United States (69, 91, 95, 96, 103), and distribution of four molecular types among other ethnic groups, especially Asians, should be further investigated in future studies.

**Table 4:** Proportions of the four molecular subtypes of EOVC.

Reference	Regions	POLE mut	MMRd	TP53 abn	TP53 wild-type
Kramer (96)	Canada and European centers	18/511 (3.5%)	70/511 (13.7%)	49/511 (9.6%)	374/511 (73.2%)
Leskela (95)	Spanish	8/166 (4.8%)	29/166 (17.5%)	19/166 (11.4%)	110/166 (66.3%)
Hollis (69)	Britain	7/112 (6.2%)	20/112 (17.9%)	27/112 (24.1%)	58/112 (51.2%)
Cybuslka (103)	America	1/36 (2.8%)	7/36 (19.4%)	6/36 (16.7%)	22/36 (61.1%)
Parra-Herran (91)	Canada	7/72 (10.0%)	6/72 (8.3%)	17/72 (24%)	42/72 (58.3%)

mut, mutation; d, deficiency; abn, abnormal.

## Tumor Markers

Tumor markers including serum CA125 and HE4, are reportedly useful for predicting malignancy in patients with pelvic masses. Few studies have focused on the use of tumor markers in the preoperative diagnosis of EOVC. A case-control study demonstrated levels of serum CA19-9, sialyl Lewis-x antigen (SLX), carcinoembryogenic antigen (CEA), and lactate dehydrogenase (LDH) in EOVC are more likely to be higher compared with CA125 (107), however, more clinical studies are required to confirm this.

## Metastasis

Compared with EC and HGSOC, patients diagnosed with low-grade EOVC seemingly have a lower rate of metastasis. However, this phenomenon is not an absolute event and clinically, there are still some people whose tumors metastasize. With regard to the synchronous discovery of EOVC and EC discussed previously, there is an ongoing controversy concerning if there are two independent primary tumors or if there has been metastatic formation. Generally speaking, metastatic of cancer generally indicates advanced disease or a very poor prognosis, while synchronous EOVC and EEC display the opposite, with limited tumors and a surprising prognosis (108, 109). Recently, Jennifer et al. revealed that in low-grade EEC, isolated ovarian metastases were not found, however, the incidence of ovarian metastases in patients with high-grade EEC ranged from 2~3% (110). Whether the metastatic characteristics of EOVC are the same as those of EEC is worth further study. A retrospective study supported by several other papers (111, 112) found significantly higher ER positivity, but not PR positivity in EOVC without peritoneal metastases compared with cases with peritoneal metastases (93% vs 59%), suggesting ER positivity may be negatively associated with peritoneal metastases in EOVC (113).

In addition, there may be a relationship between microbial abnormality and cancer as mentioned above. In a mouse model of OC, the frequent use of antibiotics can lead to microbial dysbiosis, with a final outcome of accelerating the development and metastasis of OC (114).

## Treatment and Potential Therapy Strategies

The heterogeneity of EOC makes its treatment a challenge (115). Adjuvant therapy is universally dependent on grade and stage rather than histological type, consequently, it is necessary to implement the most appropriate treatment depending on the histological type of EOC (100).

According to the latest National Comprehensive Cancer Network (NCCN) guidelines (116), it is recommended that patients with low-grade (grade 1) EOVC should be followed these suggestions, observation is encouraged for stage IA/IB patients, similar to LGSOC; for patients with stage IC, observation or intravenous platinum-based therapy seems preferable; additionally, for women with high ER/PR expression of tumor cells, options to use hormonal therapy (tamoxifen, aromatase inhibitors); and patients with stage II-IV could be considered to accept systemic adjuvant chemotherapy following surgery. However, the treatment recommendations for grade 2/3 EOVC are the same as HGSOC, employing paclitaxel and platinum-based chemotherapy after surgical resection as the mainstay of primary treatment. Although high-grade EOVC and HGSOC have equally high response rates to platinum-based chemotherapy, high-grade EOVC seems to develop chemoresistance easily at recurrence, indicating the significance for novel therapeutics in this subtype (115). Approximately 60% of patients with EOVC may exhibit potential therapeutic targets based on the available reports (2, 68).

At present, molecular typing of carcinoma is a popular topic, the purpose of which is to explore the clinical outcomes of different subgroups and further seek the most effective therapy strategy according to different molecular risk stratification. Over the past few years, molecular typing of EC has been proved to apply equally to EOVC, which provides a potential stratified therapy strategy. Although the concept of stratified treatment strategy has been proposed, specific stratified treatment plans are not raised. Furthermore, genomic analysis using targeted sequencing technology revealed clonality in synchronous EOVC and EC, which provides further clues to whether the patients of synchronous carcinoma require adjuvant chemotherapy (117, 118). ESMO-ESGO-ESTRO guidelines in 2015 provided a series of consensus on the management of EC, no adjuvant treatment is recommended for low-risk EEC patients with grade 1-2, stage I (119), similar to the NCCN guideline for early EOVC with grade 1. Whether the treatment of early synchronous EOVC and EC follows the same recommendations or not, it’s still further evaluated. Six cycles of carboplatin and paclitaxel as standard chemotherapy regime are also suitable for EC, which supported evidence for chemotherapy of advanced-stage synchronous EOVC and EC. If radiation therapy is suggested in patients with advanced synchronous ovarian and endometrial carcinoma, this could be given following chemotherapy (64). In some young EOVC patients undergoing fertility preservation treatments, endometrial sampling should be recommended to avoid a missed diagnosis of synchronous carcinoma (119).

In the previous section, we discussed the molecular characteristics of EOVC, nonetheless, therapeutic approaches targeting these molecular mutations and defects still require more clinical trials to evaluate and verify. Maintenance treatment using PARP inhibitors has a potentially important role in a significant subset of EOVC (120, 121). ARID1A mutation will also equally enhance the sensibility of tumor cells to PARP inhibitors (122), indicating that patients with ARID1A mutation are also eligible to benefit from the use of PARP inhibitors, not just limited to patients with BRCA mutation. In addition to direct cytotoxic effects, PARP inhibitors also exhibit antitumor immunity (123, 124), combination of PARP inhibitors and immune checkpoint inhibitors for the therapy of EOC have obtained positive results in some clinical trials (125). Moreover, molecular mutations in other signal pathway (PI3K/AKT/mTOR, Wnt/β-catenin/Tcf) may contribute to cell proliferation, invasion and migration, consequently, targeted inhibitors of such pathways may be able to overcome the limitations of single kinase inhibition and maybe useful in EOVC patients (126).

To change the ending of this disease, targeted therapy, immunotherapy and combination therapy are widely applied in EOC, as the new therapeutic strategies. EOC with MMR deficiency is more susceptible to the immunotherapies based on PD-1/PD-L1 pathway antibodies (127, 128), this kind of treatment may be valuable for patients with MMR deficient group of EOVC. In the same line, POLE gene damaging variants (129) and TP53 mutation (130) may also be correlated with the immunotherapeutic effect, this suggests that the combination of immune checkpoint inhibitor and certain pathway-targeted drugs may be a future direction for stratified therapy of EOVC patients. Besides, it’s reported that the DGKA-c-JUN-WEE1 signal pathway participates in the mechanism of platinum resistance in EOC patients (131), providing direction for targeted therapy to antagonize platinum resistance in the future study. Compared with effective but poorly tolerated concurrent therapy, Fang et al. found that sequential therapy with PARP and either WEE1 or ATR inhibitors is effective and less toxic in the study of EOC model (132). A phase II study published in 2016 showed that AZD1775, a WEE1 inhibitor, could sensitize the efficiency of carboplatin to some extent in the treatment of TP53-mutated EOC (133). Response to the immune checkpoint inhibitor could be associated with alteration of copy number and immunotherapy resistance may be apparent in EOC with high copy number alterations (134–136), but more research in the field is required in the future. Currently, the clinical trials of combination of immunotherapy with other treatment options are ongoing, combined therapies have collaborative effects in contrast to the use of a single treatment (137).

Many patients with low-grade EOVC have high expression of both ER and PR. Evidence for benefits of anti-estrogen treatments in ER-positive patients is accumulating, especially at a state of low tumor burden after primary chemotherapy or in the maintenance phase between chemotherapies (138). However, more studies are warranted to seek new biomarkers to help identify estrogen-responsive cancers more precisely (139). Endocrine therapy may also be a possible effective treatment attempt for patients with EOVC who have high PR positivity and don’t accept systemic chemotherapy (93, 140, 141) following primary surgical debulking. A study using cultured primary OC cells showed that about 60% of EOVC cells are PR(+), ER(+), and the survival ability of cancer cells after application of progesterone dropped significantly (142). Several patients with high-grade EOVC or advanced EOVC were treated with endocrine therapy and obtained encouraging therapeutic effects (138, 140). Even so, there is still very limited information on the sensitivity of EOVC to hormonal therapies, and further studies are warranted.

Interestingly, extensive evidence suggests that the human microbiome plays a crucial role in influencing cancer therapy, through modulating therapeutic response to treatment and mediating treatment-related toxicity (143, 144). The possibility of altering the microbiome as a therapeutic modality has been proposed, which may improve the immune system, activate anti-tumor response, and mediate chemotherapy resistance (114); this certainly deserves further investigation.

## Prognosis and Independent Prognostic Factors

Although the prognosis of EOVC is satisfactory compared with other histological subtypes, there are still big differences present when EOVC is further classified molecularly. In the analysis of prognosis, the TP53abn group has a poor prognosis, with a 10-year disease-specific survival (DSS) of lower than 40%; whereas the POLEmut group has the best prognosis among the four molecular subtypes. The prognoses of TP53wt group and MMRd are between those of the TP53abn and POLEmut groups (91, 96, 103). Moreover, previous studies have revealed a high frequency of recurrence and death among patients with tumors of the TP53abn group and whereas none of the patients carrying a POLE-mutated tumor had recurrence or died. TP53 wild-type group generally has a favorable prognosis, but some patients in the low-stage setting who did not accept adjuvant therapy did succumb to the disease suggesting there is actually an extensive spectrum of outcomes. Therefore additional biomarkers may be essential to identify specific patients in the TP53 wild-type group, who may have additional benefit from more aggressive management (96).

The major factors that affect the prognosis in univariate analysis were reported as the following: menopausal status, FIGO staging, histological grade, lymph node dissection, ascites cytology, and hormone receptor expression. The main factors that significantly affect prognosis in multivariate analysis are grade 3 and lymph node dissection (111, 145). Among them, FIGO staging is the most significant factor. Histologic grade was not a prognostic factor among early-stage EOVC in current studies (96, 146).

In a study comparing different differentiation levels of EOVC and HGSOC, it was found that most EOVC cases diagnosed as grade 1/2 had a significantly better prognosis than those diagnosed with HGSOC, while the prognosis of grade 3 EOVC patients was not significantly different from that of HGSOC (147). This means that although the development of high-grade EOVC is rare, the poor prognosis requires attention and accurate diagnosis. For a female diagnosed with synchronous EOVC and EC, the prognosis is better than that of a single locally advanced or metastatic tumor, and the recurrence risk is lower. After comparing whether or not to chemotherapy was administered after surgery, there is still no significant difference in survival benefit in those with synchronous tumors (148), the prognosis is poor if the myometrium is invaded; while the impact of lymph node metastasis and peritoneal dissemination on survival has no statistical difference (148).

The prognostic value of many other biomarkers was also evaluated in EOVC (**Table 5**). High expression of PR and/or ER in EOVC patients has been shown in several articles as a favorable prognosis factor (93, 111, 112). ARID1A, β-Catenin, and TP53 could be used with conventional clinical and histological factors to predict the prognosis of patients with EOVC (77). Meanwhile, expression of CDX2 and nuclear β-Catenin independently or in combination appear to be positive prognostic factors (149). SATB2 expression is also an independent marker for improved progression-free survival for EOVC cases, especially for advanced patients (150). In patients with FIGO stage I~II EOVC, CTNNB1 mutations and nuclear β-catenin expression are associated with a better prognosis (disease-free survival), in contrast to a worse prognosis in EEC (56). P16 block expression in EOVC cases is more frequently found in the worse prognosis stage III/IV and grade 3 cases (151). Other novel prognostic biomarkers for early-stage EOVC have been reported, recently, an article was first to publish evidence of a connection between CECR1, KIF26B, and PIK3CA protein expression and prognosis in EOVC (152). More biomarkers should be included in the study of prognosis.

**Table 5:** Possible factors affecting EOVC prognosis.

	Possible factors
Favorable prognosis	Stage I/II or grade1/2
	ER expression and/or PR expression
	SATB2 expression
	Nuclear ARID1A positive expression
	CTNNB1 mutations and nuclear β-catenin expression
	Nuclearβ-catenin and CDX2 expression individually or in combination
Unfavorable prognosis	Stage III/IV or grade 3
	P53 abnormal
	P16-block expression
	Nuclear ARID1A negative expression (BAF250a loss)
	Negative protein expression of CECR1, KIF26B, and PIK3CA

## Conclusion and Implications for the Future

In conclusion, compared with HGSOC and other histologic types of EOC, EOVC displays distinct molecular characteristics and has a better prognosis. Persistent existence of microbial dysbiosis in the upper reproductive tract has been suggested to play a role in the development of carcinogenesis, and hence there are promises to provide unique and interesting insights and guidance into health and disease in follow–up studies. Moreover, although the prognostics significance of molecular classification remains to be demonstrated, it has important therapeutic implications in the context of the popularity of targeted therapy and immunotherapy. Thus, in females with EOVC, further individualized treatment according to biomarker and classification may be valuable to improve prognosis and quality of life.

## Author Contributions

YZ conceived the project. SC and YL reviewed the literature and drafted the article. SD and LL contributed to classifying the literature, LQ revised the manuscript. WX and YZ contributed to funding and general oversight of the project. All authors contributed to the article and approved the submitted version.

## Funding

This work was supported by National Key Research and Development Program (2018YFC1003900), the National Natural Science Foundation of China (81872110, 81902632), the Strategic Priority Research Program of the Chinese Academy of Sciences (XDB29030000), the Ministry of Science and Technology of China (2016YFC1303503), and the Fundamental Research Funds for the Central Universities (WK9110000104). The funders had no role in the study design, data collection and analysis, decision to publish, or preparation of the manuscript.

## Conflict of Interest

The authors declare that the research was conducted in the absence of any commercial or financial relationships that could be construed as a potential conflict of interest.
